# Supplementary material for: Cost–Utility Analysis of PCSK9 Inhibitors and Quality of Life: A Two-Year Multicenter Non-Randomized Study
Source: Diseases. 2024 Oct 5;12(10):244. doi: 10.3390/diseases12100244 (PMC11506820; doi:10.3390/diseases12100244)
Supplement: Supplementary file 1 [file diseases-12-00244-s001.zip › diseases-3179160 Supplementary Materials File S1.pdf]

MEMOGAL INVESTIGATORS
José Seijas-Amigo1,2,3; M.ª José Mauriz-Montero4; Pedro Suarez-Artime5; Mónica Gayoso-Rey6; Ana Estany-Gestal2;Antonia Casas-Martínez7; Lara González-Freire8; Ana Rodriguez-Vazquez9; Natalia Pérez-Rodriguez10; Laura Villaverde-Piñeiro11; Concepción Castro-Rubinos12; Esther Espino-Faisán13; Moisés Rodríguez-Mañero1,3; Alberto Cordero3,14,15; José R. González-Juanatey1,4; Adrián Paz-Couce1,2; Diego Rodriguez-Penas1,2; Begoña Cardeso-Paredes1,2,3; Ana Seoane-Blanco1,2; María Moure-Gonzalez1,2; Rita Soler-Martín1,2; Luis Margusino-Framiñan4; Ana Suarez-Rodriguez5; Marisol Rodriguez-Cobos5; Juan Rojo-Valdés5; Irene Zarra-Ferro5; Karina Lorenzo-Lorenzo6; Cristina Casanova-Martinez6; Carlos Crespo-Diz8; María Dominguez-Guerra9; María Elena González-Pereira9; María Anido-García10; Iveth Michelle Tajes-Gonzalez12; Héctor Mozo-Peñalver13; Alicia Silva-Lopez13; Jose Luis Rodriguez-Sanchez16; María Jesús García-Verde16; Francisco Reyes-Santías17; Marta Ribeiro Ferreiro1,2; Raquel Garrido1,2; Paloma Sempere5.
Institutions: 1) Cardiology Department. Complejo Hospitalario Universidad de Santiago de Compostela. Santiago de Compostela. Spain; 2) Fundación Instituto de Investigación Sanitaria de Santiago de Compostela (FIDIS). Spain; 3) Centro de Investigación Biomédica en Red de Enfermedades Cardiovasculares (CIBERCV), Madrid, Spain; 4) Pharmacy Department. Complejo Hospitalario Universitario A Coruña. Spain; 5) Pharmacy Department. Complejo Hospitalario Universidad de Santiago de Compostela. Santiago de Compostela. Spain; 6) Pharmacy Department. Complejo Hospitalario Universitario de Vigo. Spain; 7) Pharmacy Department. Complejo Hospitalario Universitario de Ferrol. Spain; 8) Pharmacy Department. Complejo Hospitalario Universitario de Pontevedra. Spain; 9) Pharmacy Department. Complejo Hospitalario Universitario de Ourense; 10) Pharmacy Department. Complejo Hospitalario Universitario de Lugo; 11) Pharmacy Department. Hospital Comarcal de Monforte. Spain; 12) Pharmacy Department. Hospital Público da Mariña. Spain; 13) Pharmacy Department. Hospital do Barbanza. Spain; 14) Cardiology Department. Hospital Universitario de San Juan. Alicante, Spain; 15) Unidad de Investigación en Cardiología. Fundación para el Fomento de la Investigación Sanitaria y Biomédica de la Comunitat Valenciana (FISABIO); 16) Pharmacy Department. Hospital de Virxen da
Xunqueira de Cee. Spain; 17) Management Department, Complejo Hospitalario Universidad de Santiago de Compostela
